# Multiple Liver Nodules Mimicking Metastatic Disease as Initial Presentation of Multiple Myeloma

**DOI:** 10.1155/2018/7954816

**Published:** 2018-05-24

**Authors:** Andrew C. Tiu, Rashmika Potdar, Vivian Arguello-Gerra, Mark Morginstin

**Affiliations:** ^1^Department of Medicine, Einstein Medical Center, Philadelphia, PA, USA; ^2^Division of Hematology and Medical Oncology, Einstein Medical Center, Philadelphia, PA, USA; ^3^Department of Pathology and Laboratory Medicine, Einstein Medical Center, Philadelphia, PA, USA

## Abstract

Multiple myeloma is a malignant clonal proliferation of plasma cells in the bone marrow preceded by monoclonal gammopathy of undetermined significance. Initial presentation of multiple myeloma as extramedullary spread in soft tissues particularly in the liver is uncommon. We report a case of a 74-year-old African American female who presented with epigastric pain, hematemesis, elevated alkaline phosphatase, and gamma-glutamyl transferase. Initial impression was peptic ulcer disease; however, ultrasound and CT scan of the abdomen showed multiple liver nodules and perihepatic lymphadenopathy suggestive of metastatic disease. Biopsy of the liver nodules showed CD138 and kappa light chain-restricted positive cells consistent with extramedullary spread of multiple myeloma to the liver. The patient achieved partial response after 6 months of treatment with Velcade, cyclophosphamide, and dexamethasone (VCD). Due to severe neutropenia from cyclophosphamide, regimen was switched to Velcade, Revlimid, and dexamethasone (VRD) which resulted to very good partial response in 1 year which eventually persisted after 4 years. No controlled prospective studies have defined the standard treatment for multiple myeloma with extramedullary spread particularly to the liver. Treatment of multiple myeloma with extramedullary disease follows guidelines for multiple myeloma.

## 1. Introduction

Multiple myeloma (MM) is a malignant clonal proliferation of plasma cells in the bone marrow preceded by monoclonal gammopathy of undetermined significance (MGUS) [1]. MM is commonly diagnosed with CRAB criteria (hypercalcemia, renal insufficiency, anemia, and bone lesions) from end-organ damage by light chain deposition, plasma cell proliferation, and interaction of the plasma cells with the microenvironment. Soft tissue involvement of MM is referred to as extramedullary myeloma (EM).

EM has been described since the 19th century with a spectrum of presentations depending on the location of the tumor most commonly in organs containing reticuloendothelial cells such as liver, kidney, skin, and lymph nodes. There were no clear clinical implications or prognostic significance at that time [2]. With advanced imaging techniques such as PET/CT scan, EMs are diagnosed promptly. In 1,003 consecutive MM patients, incidence of EM was 13%, 7% at diagnosis and 6% during follow-up [3]. In another case series, in 936 patients treated for MM, only 66 presented initially as EM with liver involvement in 21% [4]. Overall, the incidence of EM is higher at relapse than at diagnosis [3, 5].

The mechanism of extramedullary involvement by multiple myeloma has been extensively reviewed by Bladé et al. [5] *vide infra*. Multiple reports have described how EMs are associated with multiple cytogenetic abnormalities in younger patients which lead to poor survival rate and progression-free survival despite the novel agents [3, 4]. Our case report focused on an elderly patient with kappa light chain MM presenting as multiple nodules in the liver. She was diagnosed in January 2013. This report emphasized the rarity of liver involvement in MM, the presentation of MM as extramedullary involvement at diagnosis, and partial response to novel agents bortezomib and lenalidomide for five years.

## 2. Case Description

A 74-year-old African American female with past medical history of atrial flutter s/p ablation, osteoarthritis, and peptic ulcer disease s/p Roux-en-Y gastrojejunostomy initially presented with epigastric pain and hematemesis with elevated alkaline phosphatase and gamma-glutamyl transferase. Review of systems was unremarkable. Family history was pertinent for breast cancer and lung cancer of her aunt and mother, respectively. She is a 15-pack-year smoker. Physical examination was unremarkable for hepatosplenomegaly and jaundice.

**Figure 1:**
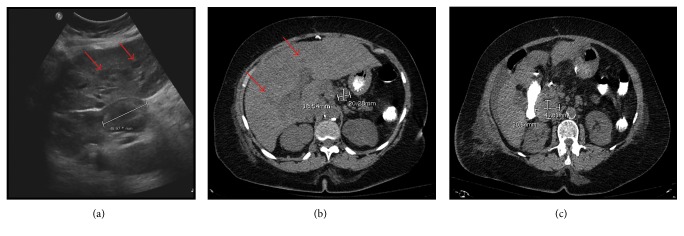
(a) Ultrasound of the liver showing 5 cm enlarged perihepatic lymph node with multiple hypoechoic nodules in the liver (red arrows); (b, c) CT scan showing multiple hypodense nodules in the liver parenchyma (red arrows) with marked enlarged perihepatic lymph nodes.

Admission labs included hemoglobin 8.3 g/dL, calcium 9.0 mg/dL, BUN 35 mg/dL, creatinine 2.0 mg/dL, total bilirubin 0.7 mg/dL, ALT 16 IU/L, and AST 21 IU/L. The elevated creatinine levels were initially attributed to hypovolemia. Esophagogastroduodenoscopy revealed gastric and jejunal ulcer while ultrasound of the hepatobiliary tract showed multiple hypoechoic liver nodules occupying at least 50% of the parenchyma and perihepatic lymphadenopathy (Figure 1(a)). CT abdomen and pelvis confirmed the innumerable liver lesions without any colonic mass and perihepatic lymphadenopathy (Figures 1(b) and 1(c)). Colonoscopy was attempted to rule out colon cancer which has metastasized to the liver but was unsuccessful. CT colonography subsequently failed to show any colonic masses or polyps.

**Figure 2:**
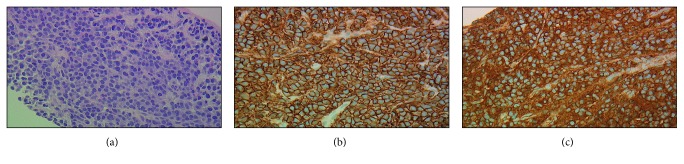
(a) Liver fine-needle aspirate (FNA) and core biopsy (400x objective) showing a monomorphic population of plasma cells with eccentric nuclei and clock-faced chromatin. Hepatocytes are not present. (b) Liver FNA immunohistochemical staining (400x objective) revealed CD138, highlighting plasma cells. (c) Kappa light surface antigen showing all plasma cells positive for stain and proving clonality.

Percutaneous biopsy of the liver nodule and perihepatic lymph node both confirmed the CD138 and kappa light chain-restricted positive cells consistent with plasmacytoma (Figure 2). There was no morphological suspicion for amyloidosis; thus, Congo red stain was not done. Labs revealed kappa light chain of 8280 mg/L, lambda light chain of 2.48 mg/L, and kappa/lambda ratio of 3338. Serum and urine immunofixation both confirmed the presence of a monoclonal kappa light chain clone and absence of a heavy chain component. The quantitative immunoglobulin levels were as follows: IgA 57 mg/dL, IgM 25 mg/dL, and IgG 366 mg/dL. There were no osteolytic lesions on skeletal survey. MRI of the brain and CT thorax with contrast were negative.

**Figure 3:**
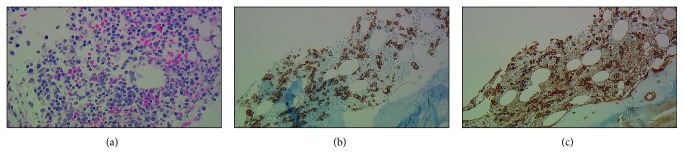
(a) Bone marrow (objective 400x) showing increased cellularity with increased scattered plasma cells intermixed with hematopoietic elements. (b, c) Bone marrow immunohistochemical staining showing CD138 and kappa light surface antigen (200x objective), highlighting scattered clonal plasma cells occupying 30–40% of total cellularity.

Bone marrow biopsy showed at least 30–40% kappa clonal plasma cells with positive CRAB criteria (hemoglobin and creatinine) confirming the diagnosis of light chain multiple myeloma (Figure 3). Fluorescence in situ hybridization (FISH) from the bone marrow showed normal (46,XX) karyotype and positive for hyperdiploidy of chromosomes 7, 9, 11, 14, and 17 with partial deletion of IgH gene. Bone marrow flow cytometry interpretation was limited due to hemodilution, processing of the sample, and clotting. There were no circulating plasma cells detected at diagnosis. According to Revised International Staging System (R-ISS) for multiple myeloma, the patient had stage III (*β*2-microglobulin level was 9.1 mg/L and LDH was 423 IU/L, without high-risk chromosomal abnormalities). This prognosticated a median progression-free survival of 29 months and overall survival of 43 months. It should be noted that R-ISS does not take EM localizations into account.

Treatment was started with CyBorD: weekly dexamethasone 40 mg, bortezomib 1.5 mg/m^2^, and cyclophosphamide 500 mg. This regimen was adopted from the multiple myeloma prognosis scoring from the R-ISS, given that the patient had R-ISS stage III with high LDH placing her at higher risk. Despite her older age, CyBorD was offered given that the patient had good baseline functional capabilities (independent and ambulatory). She also had no poorly controlled comorbid conditions.

After 6 months of treatment with CyBorD regimen, serum free light chains decreased: kappa 1690 mg/L, lambda 1.7 mg/L, and kappa/lambda ratio 994. Repeat bone marrow biopsy showed a decrease to 10% kappa clonal plasma cells, while repeat FISH showed negativity for myeloma markers such as aneusomy for chromosomes 7, 9, 11, and 17, deletion of RB1 and TP53 genes, and IgH gene rearrangement. Repeat flow cytometry showed small plasma cell clone with similar immunophenotype as the prior study. Repeat CT abdomen showed interval decrease in size of the hepatic nodules and perihepatic lymph nodes approximately 70%. In retrospect (in year 2013), this constituted a partial response according to the International Myeloma Working Group (IMWG). It should be noted that recommendations from IMWG were published on March 14, 2016 (3 years later).

Now, the patient was offered autologous stem cell transplantation; however, the patient refused, so CyBorD was continued. After 1 year, cyclophosphamide was stopped due to severe neutropenia. VRD regimen with low-dose lenalidomide 10 mg daily (21 days/28 days cycle) was started. The patient was continued on weekly bortezomib and dexamethasone. Lower dose of lenalidomide was used considering the patient's age and comorbidities. Because of severe diarrhea and rash, lenalidomide dose was further reduced to 2.5 mg daily in a stepwise manner. The patient's dexamethasone dose was reduced to 20 mg weekly due to gastric ulcer.

The patient was able to achieve very good partial response by IMWG criteria after one year of shifting regimens from CyBorD to VRD. Serum free light chains were as follows: kappa 37 mg/L, lambda 15.1 mg/L, and kappa/lambda ratio 2.45. Repeat bone marrow examination was not done; however, repeat CT abdomen showed complete disappearance of the hepatic nodules and perihepatic lymphadenopathy. Skeletal survey did not show any bone lesions.

The patient has achieved very good partial response by IMWG criteria after 4 years on the VRD regimen: kappa 65.7 mg/L, lambda 24.5 mg/L, and kappa/lambda ratio 2.68. The quantitative immunoglobulin levels were as follows: IgA 228/dL, IgM 27 mg/dL, and IgG 1067 mg/dL. The patient is presently continued on the same regimen. Unfortunately, PET/CT scan was not done at diagnosis or during the course of the disease. Currently, PET scan is the preferred imaging technique for EM.

## 3. Discussion

Soft tissue involvement of multiple myeloma particularly on the liver is rare as emphasized by the incidence described by Talamo et al. in 2,584 patients, wherein only 11 patients had liver involvement [6]. The pattern of plasma cell infiltration was described as either diffuse sinusoidal, nodular, portal, or mixed [7–11], while the mechanisms of extramedullary spread included decreased expression of adhesion molecules, downregulation of chemokine receptors, downregulation of tetraspanins, increased heparanase-1 expression, angiogenesis, and mutations in alternative or classical nuclear factor-*κ*B pathways [12]. The morphology of EMs is usually immature or plasmablastic with a shift from secreting intact immunoglobulins to free light chains (*light chain escape phenomenon*) like the case of our patient [13, 14].

Liver involvement in extramedullary myeloma is found as hypoechoic nodules on CT scan and ultrasound similar to our patient [15–17]. Rarely, it may present as hyperechoic nodules on ultrasound and hypervascular lesions on CT [18, 19]. These lesions are seen on MRI as high signal intensity on T1-weighted images and out-of-phase spoiled gradient echo [20].

The treatment of this archaic disease is still a moving target considering newer diagnostic criteria, new staging system, and more effective therapeutics [21]. Extramedullary myeloma is one of the special circumstances where treatment is not well defined due to its rarity, molecular, and proliferative heterogeneity. Initial treatment depends on risk stratification and prognostication [22]. Currently, there are two scoring systems, namely, the Revised International Staging System (R-ISS) [23] and the Mayo Stratification for Myeloma and Risk-adapted Therapy (mSMART 2.0) [24]. R-ISS comprised 3 common cytogenetic markers [del(17p) and/or t(4;14) and/or t(14;16)] while mSMART included additional molecular markers. It should be noted that both these prognostic scoring systems do not take EM localizations into account. Furthermore, mSMART has not been formally validated. For instance, in our patient, there is a discrepancy between the results of the scoring systems, wherein R-ISS is at high risk because of elevated LDH levels, while mSMART is at standard risk because of the absence of high-risk cytogenetic markers. High-risk cytogenetics is not always necessary for EM as patients without extramedullary involvement may also have high-risk cytogenetics [25]. The authors aired on the side of caution by utilizing R-ISS (high risk) in the initial management of the patient. The decision was supported by the natural history of extramedullary myeloma conferring poor prognosis as described by Varettoni et al. [3].

The current initial treatment for multiple myeloma relies on whether the patient is a transplant candidate. Velcade, Revlimid, and dexamethasone (VRD) is the standard frontline regimen, while carfilzomib replaces Velcade (KRD) if the patient has high-risk features [26, 27]. Four cycles is the duration for both induction regimens for transplant-eligible patients while 12–18 cycles is the typical duration for transplant-ineligible patients [21, 22]. For high-risk patients, carfilzomib- or bortezomib-based maintenance is utilized for 2 years after the initial treatment. Multidrug combinations such as VDT-PACE for 2 cycles (Velcade, dexamethasone, thalidomide-cisplatin, doxorubicin, cyclophosphamide, and etoposide) can also be utilized for multiple extramedullary myelomas prior to autologous stem cell transplantation (ASCT) or after aggressive relapse [28, 29]. This is usually followed by bortezomib maintenance.

Ideally, carfilzomib should be utilized in the initial treatment in our patient due to high-risk features; however, this was not yet available in 2013. Instead, CyBorD also known as VCD regimen was used [30, 31]. Currently, VCD is utilized for patients who are frail, ≥75 years old, and at intermediate risk [21]. Due to toxicity from cyclophosphamide, the authors chose to shift to VRD regimen [31, 32], which unexpectedly deepened the response from partial response to very good partial response after 1 year [33]. To date, the role of the continuous therapy with 2 different regimens is unclear. The choice of the continued VRD regimen was balanced between the wishes of the patient refusing transplant, elderly age, multiple controlled comorbidities, the toxicity of the previous regimen, the improved response with the current regimen, and the toxicity of the current regimen.

The addition of lenalidomide (Revlimid) may have been responsible for the improvement in response as documented by a few case reports. Xie et al. successfully treated secondary multiple myeloma with extramedullary liver plasmacytoma in a renal transplant patient with RCD regimen (Revlimid, cyclophosphamide, dexamethasone) [34]. Similarly, Felici et al. utilized the RCD regimen on a patient with bilateral retro-orbital localization [35]. In two patients with bortezomib-resistant extramedullary myeloma, Revlimid and dexamethasone (RD) regimen was an effective treatment according to Ito et al. [36]. CRVD (cyclophosphamide, Revlimid, Velcade, dexamethasone) was able to attain radiologic partial response in a patient with hepatic extramedullary disease as reported by Saboo et al. [37]. Bortezomib (Velcade) was originally observed to be efficacious against EM; however, these reports suffered from few sample sizes without adequate controlled trials [38, 39]. Velcade and Revlimid may have synergistic effects which potentially explain their efficacy [40]. The VDT-PACE regimen may not be an option for the patient due to potential toxicities and decreased quality of life.

Defining the best therapeutic regimen to manage the development and progression of extramedullary myeloma remains a challenge. What is certain is that newer agents can improve outcome [41–47]. For every regimen that is started, continued monitoring of response to treatment is warranted. [18F]-FDG PET/CT is the recommended imaging modality especially for hepatic lesions of extramedullary myeloma [37].

## 4. Conclusion

The approach to a patient with multiple liver nodules is a diagnostic challenge. Once imaging and diagnostic tests ruled out common causes of multiple liver nodules such as primary hepatobiliary cancer, metastatic disease from colorectal cancer, and infection, we can then pursue investigating other infiltrative diseases to the liver such as hematologic malignancies. The presence of anemia, kidney dysfunction, and altered albumin-globulin ratio made the authors suspect multiple myeloma. Our patient had no specific physical examination findings that would suggest a hematologic malignancy such as hepatosplenomegaly, lymphadenopathy, and skeletal pain. Furthermore, there were no specific imaging features for extramedullary myeloma involvement of the liver. Ultimately, biopsy was done to confirm the diagnosis.

EMs are not always associated with high-risk cytogenetic abnormality. Because of the patient's older age, multiple comorbidities, higher *β*2-microglobulin levels, kappa light chain monoclonal gammopathy, extramedullary involvement of the liver, and no high-risk cytogenetics, the risk stratification and treatment options become more complex and must be individualized. There are no clear prognostication factors as to which patients with multiple myeloma have higher risk of presenting as extramedullary disease due to infrequent incidence of EM on diagnosis and the molecular and cytogenetic heterogeneity of MM. The challenge is that most patients who are newly diagnosed have no known risk factors and risk stratification must be continued throughout therapy as these dictate changes in the management. Close follow-up is therefore warranted in this patient to monitor relapse and end-organ damage from MM.
